# Resource Bricolage for Inclusive Employment: A Case Study of Social Entrepreneurship to Support Parents of Individuals with Intellectual Disabilities

**DOI:** 10.3390/bs16020274

**Published:** 2026-02-13

**Authors:** Zengke An, Qianru Zhang, Yi Liu, Jingwen Lv

**Affiliations:** 1 School of Business Administration, Zhongnan University of Economics and Law, Wuhan 430073, China; anzengke@zuel.edu.cn; 2 School of Business, Macau University of Science and Technology, Macau, China; qrzhang@must.edu.mo (Q.Z.); 2230008989@student.must.edu.mo (J.L.); 3 School of Innovation and Entrepreneurship, Shandong University, Qingdao 266237, China

**Keywords:** family-driven social enterprises, intellectual disabilities, resource bricolage, inclusive employment

## Abstract

Supporting employment for people with intellectual disabilities is essential for their social inclusion and psychological well-being. Previous studies have explored how social enterprises facilitate employment for this group. However, relatively little attention has been given to family-driven social enterprises, particularly the behavioral motivations and mechanisms through which parents create inclusive work opportunities for their adult children. This exploratory single-case study investigates why and how parents engage in social entrepreneurship to support individuals with intellectual disabilities. Findings show that parents dynamically mobilize human and material capital, driven by both egoistic and altruistic motives. Meanwhile, the enterprise gradually evolved from an informal initiative into a more structured organization. Employment inclusion emerged as the primary outcome, enabled by knowledge acquisition and capacity building, with social empowerment as a broader benefit. The study contributes by identifying dual parental motivations, extending resource bricolage to family-driven social entrepreneurship, and reconceptualizing bricolage as a strategic management of human and material capital.

## 1. Introduction

The global push for social inclusion emphasizes the need for people with intellectual disabilities to contribute meaningfully to society, not just as recipients of care (Arnold et al., 2025). Such a shift is part of a broader movement to address the marginalization of vulnerable populations worldwide. Within this context, the present study focuses on a single family-driven social enterprise, JIEXIN Laundry, as an illustrative case to explore how inclusive employment and participation mechanisms can be operationalized. JIEXIN Laundry located in Nanjing, China, was established by a couple motivated by their long-term care needs of their adult child with an intellectual disability. Over the past two years, the enterprise has gradually expanded beyond the family sphere into a community-oriented initiative. With the involvement of other families and support from both the public and private stakeholders, the initiative not only creates job opportunities but also fosters social inclusion, benefiting both the individuals directly involved and the wider community. This study does not aim to generalize findings beyond this specific case. Instead, it systematically investigates the experiences of the parents and other key participants in JIEXIN Laundry, highlighting the strategies they have employed to balance caregiving responsibilities with entrepreneurial activities. In doing so, it explores how this particular family navigated challenges such as securing resources, complying with regulatory requirements, and developing sustainable business practices.

Social entrepreneurship has attracted global attention over the past decades, presenting promising potential avenues to improve employment and social inclusion for marginalized groups, including people with intellectual disabilities. Specifically, some researchers examine social enterprises as vehicles for employment and social inclusion for people with intellectual disabilities, with an emphasis on organizational forms, employment outcomes, and policy contexts (Canestrino et al., 2020; Thoresen et al., 2018). Others focus on families of individuals with intellectual disabilities, predominantly from perspectives of caregiving, advocacy, and psychosocial support, rather than entrepreneurial practice (e.g., Gupta et al., 2020). A smaller body of research has begun to acknowledge the role of families in social enterprises, yet this work has largely remained conceptual or descriptive and has paid limited attention to how family members enact and sustain entrepreneurial activities in practice (e.g., Hirano et al., 2018). Exploring how parents of adult children with intellectual disabilities involved in a specific social enterprise case overcome these challenges can help bridge existing research gaps and offer case-based insights for informing policy and practice globally.

This research contributes to the literature by offering an in-depth, case-based examination of a single family-driven social enterprise, JIEXIN Laundry. With a specific focus on the experiences of parents, instructors and employees, this study provides a detailed account of how parents navigate the dual roles of caregivers and entrepreneurs within this particular organizational context. By tracing the processes, the analysis examines how inclusive employment and participation mechanisms are constructed. In doing so, this study extends existing research beyond outcome-oriented or normative discussions, offering process-level insights into how inclusive values are operationalized in everyday organizational practices. Founded on empirical analyses, the case-based findings clarify the specific contribution of a family-initiated social enterprise to social inclusion within its local context and enrich current understanding of social entrepreneurship involving intellectual disabilities through empirically grounded, context-sensitive analysis.

### 1.1. Employment Difficulties and Support Strategies for People with Intellectual Disabilities

People with intellectual disabilities continue to face significant challenges in the labor market, where their employment rates remain largely lower than those of non-disabled peers (Friedman, 2020). Despite various initiatives aimed at promoting employment for this group, they still encounter numerous barriers, including difficulties with workplace adaptation, psychological challenges, financial and employment burden, stigma, denial of access to education, safety concerns, and discrimination (Bialik & Mhiri, 2022; Carmichael & Clarke, 2022). These societal prejudices often lead to limited employment opportunities. Thus, overcoming such obstacles and ensuring suitable employment opportunities for people with intellectual disabilities has become an important concern that forms the broader context for the present case study. Existing studies have examined strategies aimed at enhancing employment opportunities for people with intellectual disabilities. Research highlights the importance of protecting employment rights, standardizing vocational training, and providing workplace adaptation services (Pinilla-Roncancio & Rodríguez Caicedo, 2022; Zhang et al., 2023), which have been identified as key strategies for improving employment rates and employment quality. These approaches may have contributed to the reduction in unemployment rates among people with intellectual disabilities (Burke et al., 2024; Shaw et al., 2022). However, previous strategies have largely focused on external support systems, with comparatively less attention paid to how families allocate and mobilize resources as forms of internal support. Enriching family-driven internal support, except for existing external resources, to promote the career development and social inclusion of people with intellectual disabilities remains an underexplored topic, particularly at the level of specific organizational cases.

### 1.2. Social Enterprises

Social enterprises are commonly defined as private businesses that address social issues or market failures. Some social enterprises provide employment opportunities to people with intellectual disabilities through creating flexible employment transitions and promoting social inclusion for disadvantaged groups (Caldwell et al., 2020b; Lee et al., 2024; Thoresen et al., 2018). Existing research indicates that social enterprises may offer distinct advantages for people with intellectual disabilities, including good support mechanisms as well as a balance between work and care. These factors may enhance employment participation with task breakdown (Alborno & Kalaji, 2024; Joyce et al., 2025). Meanwhile, an individual’s willingness to learn has been identified as an important factor influencing employment sustainability (Helena et al., 2023). At the same time, people with intellectual disabilities vary in their abilities and capacity, which may shape their engagement in work activities and the other forms of support required. Moreover, ethical considerations in responding to social challenges and balancing social and economic objectives have also been highlighted as central to social entrepreneurial practice (Kimmitt & Muñoz, 2018). Beyond these perspectives, social enterprise research has also adopted process-oriented and behavioral approaches, which conceptualize social entrepreneurship as a dynamic set of practices shaped by entrepreneurial motivation, learning, and resource mobilization over time (Bacq & Alt, 2018; Mair & Martí, 2006; Zahra et al., 2009). From this view, social enterprises are understood not merely as organizational forms, but as evolving processes through which social value is enacted in everyday practice.

While this body of literature offers valuable insights, it also reflects a degree of conceptual fragmentation, with studies varying in their emphasis on employment outcomes, individual capabilities, ethical responsibility, or organizational forms. To address this fragmentation, the present study relies on resource bricolage theory and adopts social entrepreneurship as a process-oriented analytical framework to examine the development of JIEXIN Laundry. Within this framework, the transformation of the enterprise from an informal, home-based family initiative into a community-oriented entrepreneurial organization is understood as a dynamic process shaped by the mobilization of both human and material capital. Specifically, individuals with intellectual disabilities acquire job-related knowledge through repeated engagement in routine work procedures, combined with ongoing processes of knowledge integration and internalization; this sustained repetition enables the gradual building and strengthening of work-related capacities. Family caregiving responsibilities are conceptualized as the central contextual condition that motivates and constrains entrepreneurial action, while inclusive employment and participation mechanisms constitute the core organizational practices through which social entrepreneurship is enacted in this case. This integrative perspective provides a coherent analytical lens for examining how employment inclusion, skill development, and social participation are organized and sustained within a specific family-driven social enterprise.

### 1.3. Work Integration Social Enterprises Within Social Entrepreneurship

Within the broader literature on social entrepreneurship, work integration social enterprises (WISEs) are commonly conceptualized as a specific organizational form that embeds employment integration for disadvantaged groups. To satisfy the diverse employment needs, WISEs targeting people with intellectual disabilities have been gradually developed. In terms of sustainable development of WISEs, in addition to focusing on the needs of vulnerable populations (Chui et al., 2019; Snipstad, 2022), the person-centered models and inclusive research practices are also valued (Gallo & Melé, 2025; Kover & Abbeduto, 2023).

WISEs prioritize ability matching and transitional support, creating comparatively stable career pathways for people with intellectual disabilities. However, most studies emphasize organizational factors, such as institutional context, social capital, and policy incentives, while relatively little attention is given to the role of founders. Specifically, the motivations of founders, resource allocation, and family commitment remain underexplored, particularly at the level of individual cases. This gap limits our understanding of how “family-driven” initiatives are enacted within specific organizational settings. Addressing these elements through in-depth analysis of a single case may provide deeper insights into how sustainable employment outcomes are pursued within a particular family-driven social enterprise.

### 1.4. Theoretical Foundation

The concept of resource bricolage, originally introduced by Levi-Strauss in the 1960s in the field of anthropology, was later adapted for entrepreneurship research by Baker and Nelson (2005). In this context, resource bricolage highlights the importance of utilizing existing resources rather than acquiring new ones, particularly in resource-scarce entrepreneurial environments (De Klerk, 2015). Subsequent research has further explored the theory of resource bricolage, focusing on the processes of organizational design, innovation, and knowledge creation (An et al., 2018; Huang & Tu, 2025). These studies investigate how entrepreneurs adeptly reconfigure existing resources to achieve their objectives despite constraints. Prior research indicates that obsolete or residual physical resources can be rendered productive through bricolage, though the effectiveness of such practices remains highly context-dependent across resource-constrained environments (Kang, 2017; Bhardwaj et al., 2024). The three most basic elements of resource bricolage are resource at hand, recombination, and making do (Baker & Nelson, 2005). Within this framework, entrepreneurs may utilize existing financial, technological, informational, policy, and social resources depending on contextual conditions. For entrepreneurial parents caring for adult children with intellectual disabilities, this often means balancing financial and prosocial goals. Given caregiving demands and limited resources, the concept of resource bricolage offers a practical alternative for mobilizing existing supports, such as family and networks, to drive entrepreneurial activities (Baker & Nelson, 2005).

The essence of resource bricolage lies in enhancing efficiency and maximizing value. Employing people with intellectual disabilities involves addressing key employment barriers such as discrimination, stigma, work environments, and employer attitudes (Jacob et al., 2023). Family-driven enterprises can effectively mitigate these barriers and optimize resource utilization by implementing person-centered support strategies. These enterprises not only promote inclusive employment but also underscore the potential of people with intellectual disabilities as valuable contributors. Existing research shows that intangible capacities of people with intellectual disabilities can become productive resources, when recombined and activated within entrepreneurial or supportive organizational settings (Pavey, 2006; Jammaers & Williams, 2023; Chui et al., 2023). Consequently, scholars advocate for reframing disability as a source of value, emphasizing capabilities rather than burdens (Maravelias, 2022; Mauksch & Dey, 2024). This shift in perspective can enhance the sustainability and impact of social enterprises while promoting a more equitable labor market.

Resource bricolage theory has accumulated a substantial body of scholarship in entrepreneurship, particularly on how entrepreneurs mobilize limited resources to foster success and how resource management can stimulate innovation within firms. Moreover, limited resources are not necessarily restrictive; under certain conditions, they can be transformed into opportunities (Baker & Nelson, 2005). However, little is known about how parents draw on resource bricolage within specific family-driven social enterprise cases to support their adult children with intellectual disabilities. Therefore, exploring the role of resource bricolage through an in-depth single-case analysis offers theoretical and practical insights. These insights clarify how social inclusion is pursued in a specific family-initiated social enterprise.

In addition, recent research on social innovation emphasizes that the development of social enterprises is not only a matter of resource mobilization, but also an organizational and relational process shaped by openness to external actors and stakeholder engagement (Kimmitt & Muñoz, 2018; Hota et al., 2019). From this perspective, social innovation emerges through the alignment of organizational practices, social missions, and interactions with communities, beneficiaries, and institutional actors. Integrating this literature allows resource bricolage to be understood not merely as an internal response to scarcity. It can also be seen as a mechanism through which family-driven social enterprises gradually structure themselves and embed innovation within broader social contexts.

### 1.5. Research Gap and Study Objectives

As shown in Table 1, this study centers on resource bricolage in social enterprises, examined within the family-based context of intellectual disabilities, to understand how families mobilize resources to create inclusive employment in practice.

Previous research on resources for people with intellectual disabilities has primarily examined employment support, social participation, and quality of life (Álvarez-Aguado et al., 2023; Gauthier-Boudreault et al., 2019; Louw et al., 2020). While these studies offer valuable insights, many adopt a single dimension perspective and provide limited insight into how families integrate resources to create social enterprises. Consequently, there is still a research gap at the intersection of caregiving and entrepreneurship, as existing research does not sufficiently examine this family-driven entrepreneurial perspective (Casteleijn-Osorno & Hytti, 2025). More specifically, limited attention has been paid to the entrepreneurial efforts of parents of people with intellectual disabilities as examined through in-depth case studies.

It is worth noting that most parents of people with intellectual disabilities face disadvantages such as limited entrepreneurial experience, limited time, and insufficient resources when creating a business for their adult child with intellectual disabilities (Malouf & Dymond, 2023). However, parents’ unique strengths, forged through years of caregiving and empathy, constitute irreplaceable entrepreneurial capital that remains undervalued (Yang et al., 2016). It is seemingly ordinary accumulations and quiet dedication that often give rise to unforeseen value and opportunities, as sometimes, even unintentional actions can bring serendipitous results. For example, by drawing on their extensive entrepreneurial experience, these parents may unlock and enhance their latent abilities. These resources boost entrepreneurial confidence and offer unique opportunities for value creation. In the context of the present case, can the challenges faced by families of people with intellectual disabilities be transformed into resource advantages? What processes enable them to shift from being passive recipients to active creators and users of resources? Exploring the motivations of parents involved in a specific family-driven social enterprise, as well as how they acquire unique resources to enhance the effectiveness of their operations, is a compelling and relevant research topic. This may offer insights relevant to the well-being of participating individuals and families, while also contributing to the growth of social enterprises.

To address this gap, this study examines the following research question: how does resource bricolage enable parents of adults with intellectual disabilities to transform caregiving-related constraints into resource advantages that support inclusive employment participation within a family-driven social enterprise? Accordingly, our research focuses on a single social enterprise founded by parents of an individual with intellectual disabilities, namely JIEXIN Laundry. In this case, the social enterprise was described by participants as supporting the transition from dependent care toward greater independence. Participants further described how both employees with intellectual disabilities and their parents gradually established connections with the external environment.

In this context, entrepreneurship and social entrepreneurship research typically revolves around three key aspects: (1) motivations—why they act; (2) management—how they act; and (3) outcomes—what happens when they act (Caldwell et al., 2020a). Given the exploratory nature of the research and the limited empirical attention to family-driven social enterprises, a qualitative single-case design was deemed appropriate for capturing the complexity of motivations, behaviors, and outcomes. To operationalize these research issues within the present case, this study seeks to achieve three objectives: first, to explore the motivations driving social entrepreneurship among parents of people with intellectual disabilities; second, to identify the essence of management in social entrepreneurship; and third, to analyze the outcomes of social entrepreneurial efforts.

## 2. Participants and Methods

### 2.1. Research Design

A qualitative single-case design was employed to explore the entrepreneurial motivations, behaviors, and outcomes of parents of people with intellectual disabilities. Case studies are well-suited for examining contemporary phenomena in real-world contexts (Suddaby et al., 2015; Yin, 1981), particularly when behaviors must be interpreted in relation to specific social environments. This study adopts a single-case design, in which JIEXIN Laundry serves as the primary unit of analysis. Purposeful sampling was employed to recruit embedded participants who occupied different roles within the same case, including founders/parents, other parents, instructors, and employees with intellectual disabilities. Rather than constituting multiple analytical cases, these participants provided complementary perspectives on a shared organizational context, thereby enhancing the depth and credibility of the case analysis. Following Siggelkow’s (2007) advocacy for cases that “crystallize theoretical tensions”, this study focused on JIEXIN Laundry, a family-driven social enterprise founded in 2023 by parents of an adult child with intellectual disabilities in Nanjing, China. JIEXIN provides vocational rehabilitation and employment opportunities for people with intellectual disabilities. Under professional guidance, employees engage in structured tasks such as sorting, cleaning, and sterilizing.

Beyond employment, JIEXIN promotes social inclusion by creating an environment where people with disabilities can develop skills, engage in meaningful work, and interact with others as contributing members of society. Through community outreach, public engagement, and profit reinvestment, the enterprise fosters a shared sense of value and belonging, helping to reduce marginalization of disadvantaged groups. Despite its modest scale, JIEXIN demonstrates how parents of people with intellectual disabilities mobilize available resources through bricolage to support employment and inclusion. Its transformation from a family-run initiative to a community-supported venture reflects a dynamic process. The single-case design enables a detailed analysis that reveals the mechanisms behind observed practices and contributes to theory-building.

### 2.2. Data Collection

Semi-structured, in-depth interviews and participant observations were conducted with the 12 participants to gather the qualitative data. After clarifying the study’s objectives, an interview outline was designed to highlight key questions, which then guided the interviewees in exploring the topics in depth. At the beginning of each interview, consent to record the responses was obtained, and transcription software was used to convert the audio recordings into text. We invited parents who are also founders to share their insights on entrepreneurial motivations, the challenges encountered, and their strategies for overcoming them. We also interviewed other parents to share their adult children’s development before and after working at JIEXIN, focusing on performance and social inclusion. Additionally, we conducted interviews with employees with intellectual disabilities to learn about their working experiences, the skills they have acquired, and any improvements in their lives. We also interviewed workplace instructors about training and rehabilitation. Each interview lasted between 30 and 60 min.

Moreover, participatory observation was used to obtain more accurate and realistic information by working with respondents and actively engaging in the daily operation of JIEXIN. One of the researchers spent one month working alongside people with intellectual disabilities at JIEXIN, which provided an immersive understanding of their daily practices and interactions. Participant observation data were recorded through systematic field notes and reflective memos. During the observation period, the researcher documented daily work routines, interactions among participants, and notable behavioral changes in written field notes. Reflective memos were written regularly to capture preliminary interpretations and emerging insights. These observation records were subsequently integrated with interview transcripts during the coding and analysis process. Participant observation refers to a qualitative method in which the researcher directly engages in the field, simultaneously participating in and observing the social processes being studied (Seim, 2024). This approach helped researchers gain a comprehensive understanding of how parents of people with intellectual disabilities achieve entrepreneurship through resource bricolage and the translation of related knowledge.

Purposeful sampling was used to select participants who can provide detailed insights into the project. This approach focused on respondents with direct experience and perspectives on the social enterprise JIEXIN. To capture diverse viewpoints, participants were drawn from different roles (Silverman, 2017), including parents, instructors, and employees. In this study, parents are family members of employees, two of whom are JIEXIN founders. Instructors guide and support employees in their daily work tasks, while employees include people with intellectual disabilities working at JIEXIN. Table 2 presents demographic information of the studied respondents.

### 2.3. Data Analysis

The data analysis aims to derive a deep structural understanding and theoretical insights from qualitative responses. It is an abstracting process that refines specific materials into general concepts and theories through a defined procedure. Discovering important “anchor points” from the data enables the authors to construct an interpretation of the observed materials. Building on Langley’s (1999) process approach, we applied an optimized three-step version of Van Maanen’s (1979) framework, integrating manifest and latent analysis.

Step one: manifest analysis. In this stage, we focused on describing the factual process from the interviewees’ perspective and constructing first-order concepts to uncover the facts. First-order coding was primarily derived from clause analysis of interviewees’ responses. Each researcher independently coded the transcripts to analyze facts. These first-order codes served as the empirical foundation for subsequent analysis in later phases.

Step two: latent analysis. In the second stage, we moved beyond factual description to interpretive explanation. This stage involved examining similarities and contrasts across first-order codes, guided by the research questions of why and how parents engage in social entrepreneurship to support individuals with intellectual disabilities. Second-order analysis involved constant comparison of first-order codes from interview transcripts and observation records across participants and data sources. Codes that reflected similar underlying meanings were grouped together and abstracted into higher-level themes. Relevant literature on resource bricolage, social entrepreneurship, and disability inclusion was consulted during interpretation to contextualize emerging themes without defining predefined coding categories. Researchers conducted this step independently to minimize bias and avoid early consensus.

Step three: collective analysis. In the final stage, all researchers integrated the independently derived first- and second-order analytical results generated in the earlier phases. Collective integration involved a systematic comparison of second-order themes, with particular attention to the conceptual boundaries, definitions, and scope. When discrepancies were identified, researchers jointly revisited the underlying data excerpts to evaluate alternative interpretations. Decisions to merge, retain, or revise second-order themes were based on empirical support across cases, conceptual distinctiveness, and coherence with the overall analytical framework. This process resulted in a consolidated set of second-order themes and an integrated theoretical structure.

To integrate the qualitative findings into a coherent analytical structure, we synthesized insights across the three stages of analysis. Linkages among motivations, resource bricolage practices, and outcome dimensions were inductively derived from the data collected. The resulting model represents an empirically grounded integration of observed processes and outcomes, illustrating why and how parents engage in social entrepreneurship to support individuals with intellectual disabilities.

To enhance the reliability and validity of the analysis, several procedural strategies were employed. Reliability was supported through independent coding and analysis in the manifest and latent stages, followed by systematic comparison and collective integration of analytical results. Validity was strengthened by repeated reference to the original data excerpts, constant comparison across participants and data sources, and the examination of alternative interpretations during the collective analysis. The above procedures enhanced the analytical coherence and trustworthiness of the findings.

### 2.4. Triangulation

Triangulation was used to address the limitations of individual data sources through systematic cross-validation. Consistency between interview and observation data was examined as a criterion of analytical reliability (Turner et al., 2017). The triangulation process focused on three analytical criteria: (1) consistency of themes across data sources, (2) alignment between participants’ responses and observed practices, and (3) explanatory coherence of interpretations across different participant roles. We systematically compared the results from interviews, observations, and literature analysis. Interview data were organized, key concepts were extracted according to a coding scheme, and observation records were categorized in detail. These two data sources were then compared with the literature review to identify similarities and differences. When inconsistencies were found, the possible causes were analyzed retrospectively, and the results were finally integrated to strengthen the reliability of the conclusions.

## 3. Results

### 3.1. Dual Motivations of Family-Driven Social Entrepreneurship

This study follows the logical chain of “Motivation-Resource Bricolage-Outcomes”. Parents of people with intellectual disabilities in JIEXIN, acting as social entrepreneurs typically, exhibit dual motivations for the resource bricolage processes. The case study revealed the coexistence of egoistic and altruistic motivations in social entrepreneurship. Although egoistic and altruistic motivations are often conceptualized as distinct, they coexist and interact dynamically in this case. They reinforce each other, forming a complementary motivational system that drives parents to transform personal concerns into collective social action. Driven by egoism and altruism, people with intellectual disabilities, family members, and enterprises undergo a gradual role transformation from caregiving to entrepreneurship. Egoism, as an intrinsic motivation, is derived from people’s desire for independent living, emotional support, and a sense of responsibility given by their families (Frielink et al., 2018). Altruism, also as an intrinsic motivation, refers to a prosocial motivation that originates within the individual but produces outward benefits for peers and the broader community. It includes the role of peer groups, the atmosphere of mutual assistance provided by the community (Lorenzo-Afable et al., 2020), and the inclusive values advocated by society (Schalock et al., 2021). In the interviews, the parents expressed their intention to start the social enterprise honestly.

“Do you know what we fear the most? It’s growing old. Every year on my birthday, I can’t sleep. I keep thinking—what will happen to her when I am no longer around? … Teachers will grow old, relatives will grow old. In the end, we will leave before our children do.”

—PT_2

“Initially, we met several families facing similar difficulties. We hoped to connect families, share experiences, and support each other.”

—PT_1

These narratives illustrate how parents’ motivations combine egoism and altruism. The fear of ageing and the uncertainty about their children’s future point to parents’ need for emotional security, which reflects egoistic motives. Meanwhile, the desire to create sustainable, inclusive employment not only for their own children but also for others demonstrates altruistic intentions that extend beyond the family unit. These dual motivations encourage parents’ transition from passive caregivers to proactive social entrepreneurs, establishing the foundation for resource bricolage in the next phase of the process. The coexistence between egoism and altruism exemplifies the behavioral complexity underlying social entrepreneurship in caregiving contexts.

### 3.2. Behavioral Processes of Resource Bricolage

#### 3.2.1. Human Capital

Human capital among people with disabilities is often structured and valued differently from that of non-disabled workers, as reflected in their differentiated positioning within labor markets (Chui et al., 2023). It is inherently intangible, and commonly understood as inherent capacities for productive engagement. Even among people with disabilities, such human capital can generate tangible effects that support entrepreneurial activities and enterprise development (Pavey, 2006; Jammaers & Williams, 2023). The entrepreneurial process heavily relies on the ability to mobilize, reorganize, and effectively utilize human capital, especially when resources are scarce. From a behavioral perspective, human capital is not merely a static set of intrinsic productive capabilities but becomes consequential only when enacted in practice. Accordingly, the following observations in JIEXIN illustrate how human capital is behaviorally activated through the participation of parents and individuals with intellectual disabilities under conditions of resource bricolage.

We found that the key human capital relied upon by parent-entrepreneurs of individuals with intellectual disabilities does not primarily derive from formal education or conventional career trajectories. Instead, it is largely grounded in lived caregiving experience accumulated over time. Such human capital includes: (1) a deep understanding of their children’s needs combined with sustained patience; (2) basic operational capabilities transferred from everyday family management practices; and (3) the ability to coordinate participants and structure work tasks based on long-term caregiving experience. These forms of human capital constitute a distinctive source of competitive advantage. In JIEXIN, parents primarily relied on resources already at hand rather than formally trained professionals. Without professional backgrounds in vocational rehabilitation, parents drew on accumulated caregiving experience to break down complex laundry tasks into simplified, repeatable steps aligned with their children’s cognitive abilities.

In addition, we also found that human capital activated among employees with intellectual disabilities in JIEXIN is manifested in two dimensions: (1) high adaptability to repetitive tasks; and (2) a strong sense of role responsibility and employer loyalty. Individuals with intellectual disabilities themselves constituted a central component of human capital bricolage: their perceived limitations (e.g., preference for routine, rule adherence, task repetition) were reinterpreted as productive strengths and strategically matched with specific work tasks. This process illustrates how existing human resources were recombined and redefined to create functional roles under resource constraints.

Based on the dual motivations, the entrepreneurial resources of parents of individuals with intellectual disabilities undergo a dynamic process of aggregation, management, and reorganization. Throughout this process, the strengths of people with intellectual disabilities and their families are fully recognized, and their potential resources are leveraged (Maravelias, 2022; Mauksch & Dey, 2024). Additionally, the role of people with intellectual disabilities has changed from semi-independent individuals to apprentices and finally to graduates, gaining the ability to live independently. Family members have evolved from caregivers to experts and, ultimately, to social enterprise entrepreneurs, demonstrating a path role transformation.

The responses from people with intellectual disabilities and the instructor elaborate on the following:

“I really enjoy working here. Being at the laundry makes me happy, and I feel much more confident than before.”

—EE_1

“I want to earn my own money, so I can buy the things I like.”

—EE_2

These narratives reveal not only practical skill gains but also meaningful psychological and identity transformations among individuals with intellectual disabilities. Through participation in real work roles, they experienced increased self-efficacy, intrinsic motivation, and a strengthened sense of social identity—key behavioral mechanisms known to enhance well-being and social functioning. From a human capital perspective, resource bricolage did more than compensate for scarcity; it activated a capability-building process that allowed both parents and participants to discover and expand their potential.

At the same time, this developmental trajectory reflects an extension of bricolage theory within family-driven social entrepreneurship. Rather than merely “making do”, parents strategically mobilized and recombined limited resources to create an enabling environment in which skill development, confidence, and social inclusion could flourish. This suggests a shift from bricolage as a reactive practice to bricolage as a transformative mechanism—one that supports long-term behavioral change and strengthens the sustainability and social impact of parent-led enterprises. Entrepreneurs typically leverage every available resource at their disposal for business ventures. Some resources may seem useless or discarded to others, but entrepreneurs can integrate and innovate with their unique experience and skills. In our research, parents effectively recognize and foster their children’s strengths, such as consistency in daily tasks, strict adherence to rules, and strong work engagement, allowing these traits to gradually transform into valuable and mobilizable human capital. This lays the foundation for subsequent resource bricolage and capability enhancement. Consistent with this pattern, prior studies have shown that workers in disability-related organizations are able to develop human capital through participation in productive activities, generating benefits for both individuals and society (Pavey, 2006). Meanwhile, these bricolage practices also reflect emerging organizational routines and increasing openness to external stakeholders, as the enterprise gradually engages families, community actors, and support networks to stabilize and expand its operations (Hota et al., 2019; Kimmitt & Muñoz, 2018).

#### 3.2.2. Material Capital

From the perspective of material capital, JIEXIN’s development illustrates how resources, such as funds, assets, and facilities, evolved from initial scarcity to gradual expansion through resource bricolage. For family-driven social enterprises, physical capital serves not only as an economic foundation but also as tangible infrastructure that provides training, employment, and long-term social integration for individuals with intellectual disabilities. Material capital refers to tangible and physical resources that can be mobilized and recombined in entrepreneurial processes. Prior studies have shown that entrepreneurs often exploit physical assets in new ways under resource constraints, such as reconfiguring existing facilities, equipment, or spaces to support business operations (Yu et al., 2019). Similarly, research on entrepreneurial bricolage demonstrates how actors transform seemingly obsolete or residual physical assets into productive resources (Kang, 2017).

Initially, the enterprise of JIEXIN operated informally, relying on basic equipment and minimal facilities, funded by family contributions. At the early stage, material bricolage at JIEXIN involved making do with insufficient physical and financial resources. The enterprise initially operated from a home-based office, and its funding partly relied on government subsidies, corporate sponsorships, social donations, income from public welfare projects, charity sales, and individual contributions. As these resources were often inadequate to meet operational needs, parents played a central role by contributing both financial support and unpaid labor. However, as the business expanded, the need for more stable funding, upgraded machinery, and larger workspaces became increasingly apparent. This adaptive utilization of material capital embodies the core characteristic of bricolage: continually seeking ways to extend limited assets based on existing resources. Moreover, different resource-constrained environments require distinct forms of bricolage solutions to address specific challenges (Bhardwaj et al., 2024). This process highlights that seemingly ordinary or neglected physical resources can become sources of new opportunity when strategically recombined, reflecting the essence of input bricolage as conceptualized by Baker and Nelson (2005).

Through continuous resource bricolage, JIEXIN has evolved from an informal initiative into a more structured and institutionalized organization, making a substantial enhancement of its material capital. Such organizational formalization reflects a typical growth trajectory observed in social enterprises, whose development is often driven by strong social missions and emerging opportunities (Misbauddin et al., 2025). As JIEXIN became more organized, parents took practical steps to stabilize their operations and gradually expand their capabilities. This also supports previous research, which indicates that by leveraging the resource advantages of people with disabilities, social enterprises can not only realize their social mission but also achieve sustainable development and growth while overcoming external difficulties (Mauksch & Dey, 2024). The process of organizational development, particularly the enhancement of physical capital through the sharing of funds, facilities, and equipment, is also reflected in the interview narratives:

“In the beginning, the start-up capital for JIEXIN came from the couple PT_1 and PT_2. Other parents gradually joined and contributed what they could. But as the organization expanded, we soon faced a funding gap and struggled to secure sufficient financial support for ongoing operations.”

—PT_3

“JIEXIN plans to open several branches in the future, and the existing outstanding employees will be promoted to team leaders.”

—IR_4

“If JIEXIN Laundry could be positioned as a replicable brand model and other social enterprises invited to join, it would greatly benefit small-scale organizations like ours.”

—PT_4

Overall, the interview narratives show that resource bricolage was not only a way to keep the enterprise running but also a process of collective growth. What began as a small, self-funded effort gradually developed into a more capable and recognizable organization. This progress was supported by shared commitment and steady, meaningful contributions from different families. As JIEXIN grew, caregivers also started to imagine new possibilities for the future. This development reflects more than organizational change; it marks rising confidence, empowerment, and hope among families who had once felt isolated but can now see a future they are building together.

### 3.3. Outcomes of Family-Driven Social Entrepreneurship

Consistent with the proposed process model, employment inclusion emerged as the primary outcome of parental social entrepreneurship. Knowledge acquisition and capacity building functioned as enabling mechanisms through which individuals with intellectual disabilities gradually achieved stable work participation, while social empowerment appeared as a broader spillover effect beyond the enterprise.

#### 3.3.1. Knowledge Acquisition

In the case study of JIEXIN, knowledge acquisition emerged as an important enabling outcome of family-driven social entrepreneurship for individuals with intellectual disabilities. Previous studies have largely focused on conceptualization of learning disabilities and formal educational strategies for intellectual disabilities (Cluley, 2018). Concerns regarding knowledge acquisition for people with intellectual disabilities have ranged from methods and strategies for educating them to exploring knowledge management in higher education (Almuaqel, 2024; Zhang et al., 2023). In contrast, our findings indicate knowledge acquisition among individuals with intellectual disabilities in JIEXIN is not primarily formal vocational training-based, but is progressive and task-specific everyday work practices within a family-driven social enterprise.

More specifically, we found that positive changes in people with intellectual disabilities and their families resulted in tacit knowledge within the personal domain. Individuals with intellectual disabilities gain contextual understanding of work tasks and routines, reflecting the early development of competence. In the context of the laundry enterprise, knowledge refers to employees’ understanding of routine work procedures, including sorting, pre-treatment, washing, drying, ironing, packing, and delivery. Such procedural knowledge was primarily acquired through observation and repeated guidance from instructors during daily work activities. In group activities, instructors guide people with intellectual disabilities to share and externalize their learnings, while also discussing solutions to communicate challenges. The integration of family resources plays a critical role in the knowledge acquisition process for people with intellectual disabilities (Wang et al., 2025). The results of knowledge acquisition were also confirmed in our interview data from an employee and an instructor:

“There are different ways to fold towels. I can fold them all, and I am very good at it. I can go shopping on my own. And I also can make delicious dumplings for my mother.”

—EE_3

“They can classify towels of different colors from different stores. Each time the towels are washed or collected, they count how many of each type there are, and they can identify which store the towels come from even better than instructors.”

—IR_3

These interview records illustrate how knowledge acquisition is manifested through task performance. After all, people with intellectual disabilities sometimes face challenges in employment, in terms of both job adaptation and socialization, and previous education and employment models may not adequately meet their needs. We found that adopting a progressive knowledge acquisition model for family-driven entrepreneurial social enterprises has positive implications for people with intellectual disabilities. It emphasizes the progressive acquisition of personal knowledge, which aligns better with the physical and mental development of people with intellectual disabilities. This finding is consistent with Bakker and McMullen’s (2023) claim that unconventional entrepreneurs contribute to the knowledge transfer. Similar findings have been observed in other social enterprises, where individuals with intellectual disabilities acquire knowledge that supports the initial development of competence through meaningful work, mentorship, and customized employment support (Hutchinson et al., 2021; Smith et al., 2018).

#### 3.3.2. Capacity Building

The progressive model of innovation, which spans knowledge acquisition to employment, helps people with intellectual disabilities build self-confidence and enhance their working skills. When parents start a business for their adult children with intellectual disabilities, they need to use resources such as manpower, materials, and experience to improve their ability to integrate resources (Malouf & Dymond, 2023). In this process, parents realize the outcomes of entrepreneurship on the one hand. On the other hand, the knowledge acquired by people with intellectual disabilities at an early stage can be developed into a working capacity. Continuous integration and internalization of knowledge provide a valuable opportunity for people with intellectual disabilities to enhance their capabilities. Along this path, the gradual integration of people with intellectual disabilities into society is an altruistic process that enables enterprises to advance public welfare development. From home to business, this is a process of empowerment for both parents and their adult children with intellectual disabilities.

The findings further indicate that knowledge and skills play distinct but complementary roles. Knowledge development refers to understanding work procedures, task sequences, and operational routines, while skill development is reflected in the ability to perform tasks independently, improve efficiency, and adapt to workplace demands. At the individual level, people with intellectual disabilities transform acquired knowledge into practical working skills through repeated participation. At the family level, parents develop organizational knowledge and integration skills related to coordinating resources, structuring tasks, and managing daily operations. As one interviewed employee and one instructor stated:

“Our efficiency has significantly improved, and we are receiving more orders than before.”

—EE_4

“We learned the whole process ourselves first—how to run the laundry, how to use each machine, and even the proper way to fold towels. After that, we thought carefully about how to break the steps down so the children could understand and learn at their own pace. Through repeated practice, they gradually mastered the skills and became more confident in their work.”

—IR_2

It is worth noting that both parents’ business skills and their adult children’s career skills were enhanced. Resource bricolage requires parents to flexibly reorganize existing resources (e.g., social networks, non-traditional funding, and local manpower) under limited conditions, which is reflected in the ability to “integrate”. Meanwhile, through participation in daily activities (e.g., simple production, social interaction), people with intellectual disabilities can apply their established abilities to life and production in a supportive environment. Prior studies on social enterprises similarly show that work participation supports capacity building by enabling people to translate acquired skills into socially meaningful contributions (Gallo & Melé, 2025; Meltzer et al., 2018).

#### 3.3.3. Employment Inclusion

Building on the knowledge acquired and capabilities strengthened through the resource bricolage progress, participants gradually moved toward more stable and meaningful forms of work participation. This marks the transition from individual growth to broader employment inclusion as the focal outcome, through which earlier gains in knowledge and capacity are consolidated into stable work participation. Family-driven social enterprises create a supportive environment that bridges the gap between home-based learning and participation in the wider labor market. These tasks are tailored to the strengths of individuals with intellectual disabilities, enabling them to develop working skills. Instead of being directly placed into competitive employment, participants develop work habits through repeated engagement. This transitional adaptation allows them to gradually access work integration at their own pace, in a manner that aligns with their cognitive abilities.

“My son used to avoid social activities. Now he wakes up early because he wants to go to work. It makes him feel needed.”

—PT_1

“He now introduces himself by saying he works at a laundry shop, and he can clearly describe the washing procedures and different towel-folding methods. It shows how proud he is of his work and how much his communication skills have grown.”

—IR_1

This evidence illustrates that the employment pathways observed in this study reflect participatory approaches advocated in disability research and practice. JIEXIN did not impose a preset training model; instead, it created an environment where individuals with intellectual disabilities could learn through practice. This progressive action learning approach enabled individuals to develop working skills, communication abilities, and self-confidence in a real rather than simulated environment. As previous research has indicated, a participatory action-oriented approach is crucial for employment inclusion (Shaw et al., 2022). Moreover, the experiences of people with intellectual disabilities at JIEXIN similarly reflected a model of progressive action learning processes in context. In this sense, our findings expanded existing understandings of employment pathways for people with intellectual disabilities. While traditional models emphasize supported employment, our results show that family-driven social enterprises, as an intermediate platform, enhance work participation in a more participatory manner. Similar to other social enterprises, the case of JIEXIN shows that social enterprises can expand access to inclusive employment for people with intellectual disabilities (Babikian & Hamdani, 2023; Hafsteinsdóttir & Hardonk, 2023). Therefore, family-driven social enterprises promote employment inclusion for people with intellectual disabilities through participatory and progressive learning processes.

#### 3.3.4. Social Empowerment

With the realization of employment inclusion, the impact of resource bricolage extends beyond individual gains to broader levels of social empowerment (Ghalwash & Ismail, 2024). Social empowerment in this study emerges as a cumulative outcome of prior processes, linking employment inclusion at the individual level to empowerment effects at the family and community levels. People with intellectual disabilities are beginning to play socially recognized roles in their communities, which not only enhances their sense of belonging but also increases public recognition of their abilities. This shift reflects a conversion from individual adaptation to collective empowerment, where work participation enables individuals to make meaningful contributions to society. Meanwhile, families also experience tangible forms of empowerment.

At the family level, empowerment is reflected in increased psychological security, reduced caregiving burden, and the formation of peer support networks, which together mediate the transition from individual work participation to broader social recognition. Daily pressures ease as parents gain a greater sense of psychological security, knowing that sustainable arrangements for their children are gradually becoming possible. And supportive networks form among families, reducing feelings of isolation and creating shared spaces for problem-solving.

At the community level, the accumulation of individual and family-level changes contributes to a shift in social perceptions, whereby people with intellectual disabilities are increasingly recognized as contributors rather than dependents. The visible progress of JIEXIN serves as a demonstrable success case, offering a practical model that inspires neighboring families and signals the broader potential of family-driven social enterprises. The following interview illustrates the positive outcomes:

“At first, we only hoped our children could adapt and find a safe place to spend the day. But now, when neighbors tell us how reliable they are at work and how much they appreciate what JIEXIN does for the community, we feel proud.”

—PT_2

“After seeing how well JIEXIN runs, other parents have asked us how they can start something similar. It feels like our experience is giving directions to more families.”

—PT_1

Overall, these findings suggest that resource bricolage goes beyond simply supporting business survival; it actively enhances social empowerment for individuals, families, and communities. By creatively mobilizing limited resources, families create an environment where individuals with intellectual disabilities can be recognized as capable contributors (Suarez-Balcazar et al., 2023). This process also strengthens parents’ confidence and social connections, reducing long-term feelings of isolation. In turn, these collective benefits foster a more inclusive community atmosphere, demonstrating that family-driven resource patchwork can generate broader social value. To enhance conceptual clarity, social empowerment is theorized as the integrative outcome of family-driven social entrepreneurship, toward which knowledge acquisition, capacity building, and employment inclusion progressively orient.

### 3.4. Conceptual Model

**Figure 1:**
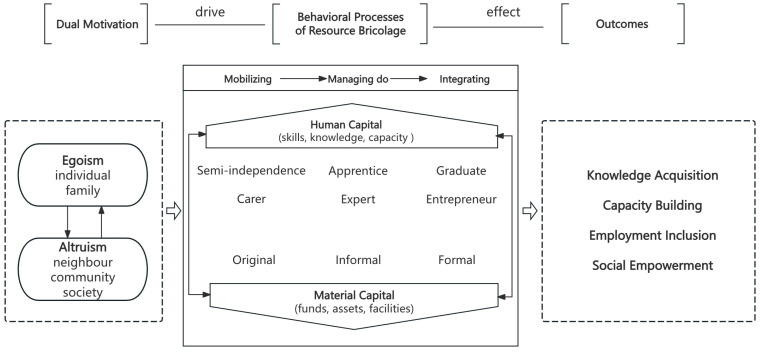
Empirical process model derived from the qualitative analysis. Note: Large arrows illustrate the primary process flow, smaller arrows indicate internal linkages, and dashed boxes denote conceptual groupings.

Figure 1 presents the conceptual model derived from the empirical findings of this study. The model shows how dual parental motivations, resource bricolage, and outcome generation are connected within the context of a family-driven social enterprise, JIEXIN.

At the motivational level, the model highlights the coexistence of egoistic and altruistic motivations. Egoistic motivation is closely tied to parents’ concerns for their own children and families, such as long-term security, independence, and continuity of care. Altruistic motivation reaches beyond the family and includes support for other families, the local community, and broader values of inclusion and social responsibility. In practice, these two forms of motivation are closely intertwined and together drive parents’ decisions to initiate and sustain social entrepreneurial activities.

Guided by these dual motivations, parents engage in resource bricolage by drawing on both human and material capital. Human capital in this case is mainly developed from long-term caregiving experience, not from formal entrepreneurial training. For parents, this includes a deep understanding of their children’s needs, sustained patience, everyday organizational skills, and the ability to coordinate participants and structure work tasks. For individuals with intellectual disabilities, human capital is reflected in their adaptability to repetitive and structured tasks, as well as a growing sense of role responsibility and commitment to the workplace.

Material capital was largely mobilized through family-based resource bricolage. Families pooled what they had, including small amounts of funding and basic physical resources, and this formed the foundation of the enterprise. The business initially operated from a home-based workspace. External funding, such as government subsidies and donations, later supplemented these family resources. Material capital inputs supported everyday operations, training, and the gradual move toward a more formal organization.

The model distinguishes four related outcomes that emerge from this process. Employment inclusion is treated as the primary outcome, reflecting sustained participation in meaningful work roles and everyday social interaction within the enterprise. Knowledge acquisition and capacity building support this outcome by helping individuals develop skills, build confidence, and establish stable work habits. Social empowerment appears as a broader and more long-term outcome, extending beyond the enterprise to families and the surrounding community through greater recognition, reduced isolation, and stronger social connections.

## 4. Discussion

### 4.1. Theoretical Contribution

This study extends resource bricolage theory to family-driven social entrepreneurship for people with intellectual disabilities (Bakker & McMullen, 2023; Malouf & Dymond, 2023). It offers empirically grounded insights into how inclusive employment was pursued within the specific context of a single family-driven social enterprise. Our research makes three major contributions to the concept of resource bricolage.

First, we identify two entrepreneurial motivations, egoistic and altruistic, that drive resource bricolage in family-driven social entrepreneurship for individuals with intellectual disabilities. Our focus extends beyond the motivations of parents starting enterprises for their adult children’s needs to include benefits for peers and broader social values. Previous studies have highlighted both self-oriented motivations and other-oriented motives as key drivers of social entrepreneurship (Ruskin et al., 2016). We argue that parents initially pursue egoistic motives to address individual needs, such as enhancing life skills, ensuring survival opportunities, and fostering interpersonal relationships. As these needs are met, altruistic motivations emerge, leading to benefits that extend beyond the family to peers and a wider community. As noted by Carsrud and Brännback (2011), entrepreneurial motivation can directly influence entrepreneurial behavior. Driven by love and responsibility, parents actively mobilize personal and social resources to support their adult children’s employment and integration, aligning with broader findings in prior research (Gupta et al., 2020).

Second, the theoretical contribution of this study lies in its extension of resource bricolage theory to the underexplored area of parental entrepreneurship for individuals with intellectual disabilities. This expansion enriches the literature at the intersection of entrepreneurship and caregiving (Casteleijn-Osorno & Hytti, 2025). While resource bricolage theory has been widely applied in general entrepreneurial contexts (Baker & Nelson, 2005) and increasingly to social enterprises (Hota et al., 2019; Kwong et al., 2017), its explanatory power remains limited when addressing emotionally driven motivations. To more accurately capture the resource allocation behaviors of parents of people with intellectual disabilities in social entrepreneurship, this study highlights an empowerment-oriented commitment. It emphasizes that when resources are constrained, actors motivated by deep emotional drives strategically allocate the most valuable resources to achieve specific goals. In social entrepreneurship research, resource bricolage theory is often employed to explain behaviors in resource-limited contexts. Baker and Nelson (2005) proposed that entrepreneurs “make do with what is at hand” to discover opportunities for survival and innovation amid uncertainty and scarcity. This theoretical framework has been widely used to explain non-profit entrepreneurship, small enterprise development, and the entrepreneurial behaviors of marginalized groups (An et al., 2018; Huang & Tu, 2025). The concept of empowering commitment, as an extension of bricolage characterized by flexibility and adaptability (Hsieh et al., 2019), underscores a sustained emphasis on person-centered approaches. This aligns with recent findings and contributes to the development of robust and integrated life projects for people with intellectual disabilities (Carvalhais et al., 2023).

Third, this study enhances the understanding of resource bricolage by emphasizing that it involves not only making do with available resources but also actively managing both human and material capital. In our case, despite the parents’ lack of entrepreneurial experience, they created an environment of irreplaceable compassion that enabled the person with intellectual disabilities to develop the ability to live and work. This reflects the concept of novice–specialist effectual entrepreneurs, who, despite lacking formal entrepreneurial experience, can cope with uncertainty by relying on the practice-based knowledge they possess (Bao et al., 2024), and extends prior work on resource management in constrained contexts (Baker & Nelson, 2005; Hota et al., 2019). Importantly, in relation to promoting empowerment (Suarez-Balcazar et al., 2023), our findings are consistent with research that highlights the value of community-based empowering settings (Madyaningrum et al., 2022). The family-driven social enterprise in this study extends “setting” by demonstrating that empowerment grows not from assuming uniform abilities, but from creating supportive environments where appropriate resources help individuals realize their unique potential. Through the discussions in this study, resource bricolage enables parents to enhance the inherent potential of their adult children with intellectual disabilities and promote their social inclusion (Mauksch & Dey, 2024). This process also resonates with social innovation perspectives that conceptualize the development of social enterprises as an organizational and relational process shaped by stakeholder engagement and openness to external actors (Kimmitt & Muñoz, 2018; Hota et al., 2019).

### 4.2. Practical Implication

#### 4.2.1. For People with Intellectual Disabilities

From a knowledge learning perspective, individuals with intellectual disabilities undergo a process of transformation that includes socialization, externalization, combination, and internalization. These processes occur in different contexts, within families, peer groups, enterprises, and society. Their knowledge evolves from basic survival skills to broader competencies. In terms of capacity enhancement, people with intellectual disabilities contribute to their families, enterprises, and society by internalizing newly acquired knowledge and acquiring corresponding skills. The distinction between explicit knowledge (know that) and tacit knowledge (know how) offers particular relevance. Tacit knowledge is often developed through informal interactions and experiential learning (Farrington et al., 2015) and plays a crucial role in the growth of people with intellectual disabilities within social enterprises. Such knowledge not only strengthens their participation in enterprise activities but also supports their broader inclusion in society.

#### 4.2.2. For Family Entrepreneurs

Enterprises provide a vital platform for the gradual social independence of people with intellectual disabilities. While strengthening vocational abilities, they should also attend to the spiritual growth of participants. Parents who transition from caregivers to social entrepreneurs need several key competencies. First, they must be adept at recognizing and mobilizing available resources, including human, material, and social networks. For instance, the emotional support and responsibility of family members, combined with community mutual aid, can facilitate enterprise development. Second, effective entrepreneurs demonstrate flexibility in resource integration. They reorganize social networks, leverage non-traditional funding, and adaptively reconfigure resources, including the workforce of people with intellectual disabilities, to meet evolving market and organizational needs (Thoresen et al., 2018). Third, they should value tacit knowledge and encourage knowledge-sharing. Parents, in particular, are well-positioned to identify the strengths of people with intellectual disabilities and to design tailored training approaches that maximize their potential.

#### 4.2.3. For Policymakers

By integrating the knowledge and capacity of people with intellectual disabilities, policymakers can adjust policies to better address the needs of their families at various stages. Inclusive policy support is a critical starting point in the transition from learning to employment (Burke et al., 2024). At the transition from the personal to group field, governments can incentivize families to engage in collaborative projects through financial support and policy initiatives. At the stage from the group to the enterprise field, favorable conditions for social entrepreneurship should be provided, such as low-interest loans, employment guidance, and volunteer service support. At the stage from the enterprise to the social field, policymakers can strengthen sustainability by offering accreditation mechanisms that validate social enterprises. To further consolidate these efforts, governments should develop integrated information platforms such as online databases that combine knowledge resources, service information, and policy guidance. Through these measures, policymakers can help transform family-driven initiatives into sustainable social enterprises, supporting the long-term inclusion of people with intellectual disabilities.

### 4.3. Limitations and Future Research

This study provides new insights into family-driven social entrepreneurship for people with intellectual disabilities, particularly in the context of resource bricolage. Findings should be interpreted as context-specific insights from JIEXIN Laundry rather than evidence about parents of individuals with intellectual disabilities more broadly. However, several limitations should be noted.

First, the single-case study design offers a rich and contextualized understanding of how resource bricolage fosters knowledge acquisition and capability development. The findings are based on a small, purposefully selected sample, which may limit the generalizability of the results to other family-driven social enterprises or broader social contexts. Future research could broaden the scope by incorporating multiple cases or comparative groups with diverse backgrounds.

Second, while resource bricolage theory effectively explains how families mobilize limited resources, the dynamic interaction between human and material capital remains underexplored. Future research could investigate how these two types of resources interact throughout the entrepreneurial process, particularly within family-driven forms of entrepreneurship.

Third, this study primarily focuses on the early stage of family-driven social entrepreneurship and lacks continuous observation of the growth of social enterprises. A longitudinal research design would be valuable to track how family-driven social enterprises support the sustained social inclusion of people with intellectual disabilities and contribute to broader social value over time. Therefore, future research could continue to track the enterprises’ development trajectory.

## 5. Conclusions

Through a qualitative case study of JIEXIN Laundry, this research examined how parents of people with intellectual disabilities establish a family-driven social enterprise motivated by both egoism and altruism. Driven by these dual motivations, they engaged in resource bricolage, mobilizing human and material capital to generate social value. This process contributed to knowledge acquisition and capacity development among individuals with intellectual disabilities and their families, while also supporting employment inclusion as a primary outcome, and related social empowerment.

Our findings illustrate that, in this case, genuine care is not merely about assisting but about enabling autonomy—echoing the principle of “teach a man to fish.” While the initiative initially began with egoistic concerns, altruism emerged as a vital and increasingly positive driver, strengthening their sustainability. This model highlights how emotionally grounded motivations and resource bricolage interacted over time within a family-driven social enterprise.

This study provides case-based insights into how a family-driven initiative can evolve under conditions of resource constraint. The JIEXIN case demonstrates that, through the practical management of human and physical capital, families of individuals with intellectual disabilities may develop a sustainable model that integrates caregiving, employment and community participation. Care and empathy serve as the foundation for cooperation among families, service providers, and communities, fostering shared growth and inclusion. This study underscores the transformative potential of families of individuals with intellectual disabilities—not only as caregivers, but as active contributors to inclusive development. We hope these findings encourage further empirical and longitudinal research to advance the long-term growth of family-driven social enterprises for people with intellectual disabilities.

## Figures and Tables

**Table 1:** Key research streams and positioning of the present study.

Research Stream	Focus of Prior Studies	Limitations in Existing Literature	Contribution of This Study	Key References
Families of people with intellectual disabilities	Caregiving responsibilities, family burden, advocacy, psychosocial well-being, parental support strategies	Entrepreneurial practices, resource mobilization, and families’ roles as organizational founders remain underexplored	Reframes parents as entrepreneurial actors who actively mobilize family-based and relational resources to create inclusive employment opportunities	Gupta et al. (2020); Hirano et al. (2018); Malouf and Dymond (2023)
Social entrepreneurship & intellectual disabilities	Employment integration and social inclusion for people with intellectual disabilities, including work-integrated social enterprises (WISEs); organizational forms, employment outcomes, and policy contexts	Limited process-level insight into inclusive employment practices in family-driven social enterprises	Provides in-depth, process-level case analysis illustrating how inclusive employment is constructed within a family-driven social enterprise	Caldwell et al. (2020b); Canestrino et al. (2020); Gallo and Melé (2025); Thoresen et al. (2018); Zahra et al. (2009)
Resource bricolage theory	Resource scarcity, innovation, firm survival and opportunity creation	Emotionally motivated, and family-centered bricolage contexts remain largely overlooked	Extends resource bricolage theory to a family-driven social enterprise, demonstrating how caregiving-related constraints are transformed into resource advantages for inclusive employment	Baker and Nelson (2005); An et al. (2018); Bhardwaj et al. (2024)

**Table 2:** Respondents’ information description. (*n* = 12).

Code	Role	Gender	Interview Location
PT_1	Parent	F	Field study
PT_2	Parent	M	Field study
PT_3	Parent	F	Online
PT_4	Parent	F	Field study
IR_1	Instructor	F	Field study
IR_2	Instructor	M	Field study
IR_3	Instructor	F	Field study
IR_4	Instructor	F	Field study
EE_1	Employee	M	Field study
EE_2	Employee	M	Field study
EE_3	Employee	M	Field study
EE_4	Employee	F	Field study

Note: PT = Parents; IR = Instructors; EE = Employees; F = Female; M = Male.

## Data Availability

The data presented in this study are available on request from the corresponding author due to ethical and privacy considerations.
